# The Efficacy of Bisphosphonates in Preventing Aromatase Inhibitor Induced Bone Loss for Postmenopausal Women with Early Breast Cancer: A Systematic Review and Meta-Analysis

**DOI:** 10.1155/2014/625060

**Published:** 2014-03-26

**Authors:** Pooleriveetil Padikkal Anagha, Suchandra Sen

**Affiliations:** Department of Pharmacy Practice, Kovai Medical Center and Hospital, Coimbatore 641014, India

## Abstract

*Objectives*. We aim to determine the efficacy of bisphosphonates in preventing aromatase inhibitor induced bone loss (AIBL) in postmenopausal women with early breast cancer. The secondary objective was to determine the safety of bisphosphonates. * Materials and Methods*. We searched electronic databases in a time period of 1995 January to 2013 June. Random effects meta-analytical models were used; between study heterogeneity and publication bias was assessed. * Results*. A total of six eligible studies reported the BMD T score of LS at 12 months and from that 3 trials of Zoledronic acid compared the change in BMD in immediate ZOL versus delayed ZOL done with subgroups like patients with normal BMD at baseline (OR = 5.402, 95% CI = 1.329–21.959, *P* value = 0.018) and osteopenic BMD at baseline (OR = 4.008, 95% CI = 2.249–7.143, *P* value = 0.0002). Both had a significant decrease in BMD that favoured the delayed ZOL; 3 trials of risedronate and ibandronate also had a significant decrease in BMD in AIs alone group. Immediate ZOL versus delayed ZOL also showed increased risk of getting an ADR in immediate group. * Conclusion*. Third generation bisphosphonates has an effect on BMD of patients who are on treatment of AIs in breast cancer. Furthermore, the patients treated with immediate ZOL had a significantly high risk of musculoskeletal ADR's than patients with delayed ZOL.

## 1. Introduction

Breast cancer is the leading cause of premature morbidity and mortality worldwide for women. More than 800,000 women are diagnosed with breast cancer approximately, and an estimated 65% to 75% of the patients with advanced metastases will develop bone metastases during the course of their disease. Over the past few years, many studies had shown that bone metastases are common in patients with breast cancer, which resulted in significant skeletal morbidity [1].

Most breast cancers (60%) express oestrogen receptor (ER) or progesterone receptor (PR) and are responsive to estrogens for growth and proliferation. Therefore, hormone-receptor-positive breast cancer can be treated by either blocking the ER with agents such as the selective ER modulator tamoxifen, or by reducing the production of oestrogens with aromatase inhibitors (AIs) [2].

Aromatase inhibitors (AIs) profoundly lower circulating oestrogen levels by inhibiting the conversion of androgen to oestrogen, predisposing them to increased bone loss and fracture risk [3–5] while tamoxifen has a protective effect on bone loss in postmenopausal women, but all other studies consistently show that AIs are associated with superior disease control compared with tamoxifen. Apart from AIs being regarded as part of routine adjuvant therapy for postmenopausal breast cancer patients, they are being studied in combination with ovarian suppression in premenopausal breast cancer patients and even for prevention. It is therefore important to thoroughly evaluate these newer agents for side effects [6]. In postmenopausal women with breast cancer, AI causes complete suppression of aromatase activity and decrease in the level of circulating oestrogen concentrations. Therefore, the postmenopausal patients receiving aromatase inhibitors are having a high risk of bone loss which will impact their quality of life [7, 8].

Hormone-receptor-positive breast cancer in postmenopausal women is treated increasingly with aromatase inhibitors because of increased efficacy and reduced incidence of endometrial cancer compared with tamoxifen. However, aromatase inhibitor therapy increase bone turnover as a result of nearly complete oestrogen depletion, leading to increases in bone loss and fragility fractures that erode the patient's functional independence and quality of life. Management of patients with aromatase inhibitor-associated bone loss (AIBL) is currently evolving and intervention strategies are under investigation. Although no treatments are specifically approved for AIBL, bisphosphonates are currently the intervention of choice for patients with low bone mineral density or evidence of rapid bone turnover, along with adequate calcium and vitamin D supplementation and a healthy lifestyle [9].

In recent years, bisphosphonates have emerged as a highly effective therapeutic option for prevention of skeletal complications, especially in patients who have breast cancer and metastatic bone disease [10]. Bisphosphonates are analogues to pyrophosphates that bind to hydroxyapatite crystals in bone and inhibit bone resorption. The third generation bisphosphonate inhibits osteoclastic resorptive activity partly through inhibition of farnesyl diphosphate synthase and protein prenylation [11]; thereby they prevent skeletal complications.

Nowadays healthcare professionals are increasingly required to improve their practice on the best available evidence. The practice of evidence-based medicine involves a process of lifelong self-directed learning in which caring for patients creates the need for important information about clinical and other health care issues. EBM recognises that research literature is constantly changing [12]. Systematic review can be applied to these literatures with some scientific strategies to provide in an explicit fashion a summary of all studies addressing a specific question, thereby allowing an account to be taken of the whole range of relevant findings on a particular topic. Meta-analysis, which may accompany a systematic review, can increase power and precision of estimates of treatment effects [13].

We conducted a systematic review and meta-analysis to estimate the efficacy of bisphosphonates in preventing aromatase inhibitor induced bone loss and their safety in breast cancer in postmenopausal women.

## 2. Materials and Methods

### 2.1. Data Sources, Search Strategy, and Study Selection

We systematically searched PubMed and Cochrane Collaboration library in a time period from January 1995 to June 2013 using the following terms: zoledronic acid, ibandronate, risedronate, aromatase inhibitor, BMD, and breast cancer.

### 2.2. Selection Criteria

#### 2.2.1. Inclusion Criteria

- Randomised control trials published in English language,
- Studies that addressed the association between the third-generation bisphosphonates with aromatase inhibitor in breast cancer treatment,
- Studies that reported BMD *T* score after the treatment with bisphosphonate and aromatase inhibitor,
- Studies that addressed third-generation bisphosphonates (zoledronic acid, ibandronate, and risedronate),
- Postmenopausal women with early breast cancer.

#### 2.2.2. Exclusion Criteria

- Observational studies and case control studies,
- Quasirandomised trials,
- Unpublished literature,
- RCTs published in abstracts,
- Breast cancer as secondary carcinoma,
- RCTs which have to be purchased.

### 2.3. Data Extraction

Data extraction performed independently by two observers (APP and SS), with disagreements resolved by the third observer (AV). We extracted the following information: the author's name, name of each trial, the publication year, sample size, intervention, the type of bisphosphonates used, the type of aromatase inhibitor used in the treatment, and also the median follow-up time.

### 2.4. Statistical Analysis

Odds ratio with 95% CI for dichotomous variables and standard mean difference with 95% CI for continuous variables were used to assess the ADRs and BMD analysis. Heterogeneity between the studies was tested by using random effect model that was used throughout the statistical tests. We quantified the methodological qualities of studies using Jadad scores [14]. Funnel plots were used to test the publication bias and also the *P* value less than 0.05 was considered significant. Sensitivity analysis was not performed due to small number of studies. All analyses were performed by using the software Comprehensive Meta-Analysis (version 2.2.048, Biostat, USA).

## 3. Results

**Figure 1:**
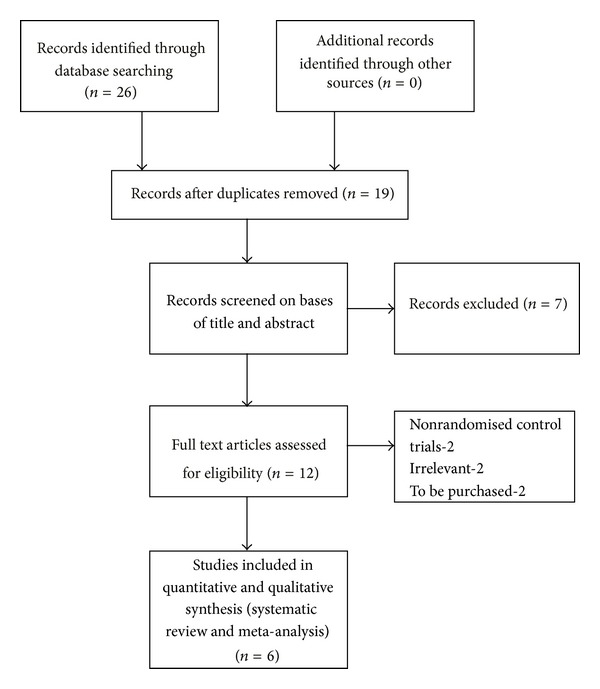
Diagram of literature search and trial selection process.

Of the 26 studies identified by database searching, 7 duplicates were removed, and 7 trials were removed on the basis of the title and abstract themselves. Finally 6 randomised control trials were eligible for the meta-analysis. All the 6 randomised control trials [15–20] addressed third-generation bisphosphonates and aromatase inhibitors treatment effect in breast cancer treatment (Figure 1). Of the six studies included, two were with risedronate and one with ibandronate while the remaining three reported zoledronic acid. In the risedronate and ibandronate trials, comparisons were made with placebo while the zoledronic acid trials were compared upfront versus delayed therapy.  Table 1 gives the details of evidence-based approach in our study and Tables 2 and 3 give the characteristics of studies included and characteristics of patients included in our study, respectively. From these studies, three of the trials reported musculoskeletal disorders of zoledronic acid in immediate and delayed treatment groups, which were used to measure the safety of bisphosphonates (Table 4).

### 3.1. Bone Mineral Density

#### 3.1.1. Delayed ZOL versus Immediate ZOL

In all the three studies (Z FAST, ZO FAST, and E-ZO FAST), the lumbar spine (LS) bone mineral density (BMD) was measured. The BMD was found to be increased in the group on immediate zoledronic acid, while in the delayed group there was a general tendency for it to decrease. In the Z FAST group, at 12 months, BMD at lumbar spine (LS) was higher in the upfront group by 4.4% (*P* < 0.0001), while in the ZO FAST and E-ZO FAST trials the same was 5.790 (*P* < 0.001) and 5.43% (*P* < 0.0001), respectively.

**Figure 2:**
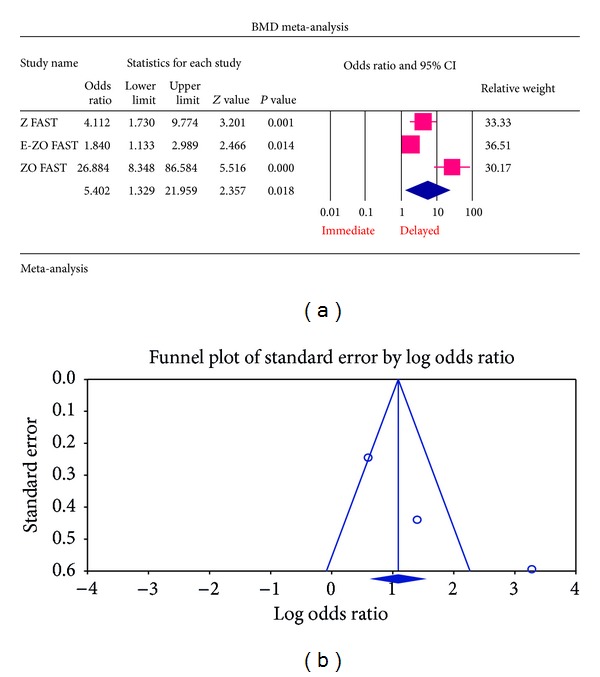
(a) Forest plot from the meta-analysis of LS BMD *T* score analysis of patients at 12 months, who had normal BMD at baseline, between immediate and delayed zoledronic acid groups. (b) Funnel plot from the meta-analysis of LS BMD *T* score analysis of patients at 12 months, who had normal BMD at baseline, between immediate and delayed zoledronic acid groups.

*(a) Patients with Normal Bone Mineral Density at Baseline*. From the BMD analysis of LS BMD at 12 month, who had normal BMD at baseline, data from the trials were pooled together to get an overall summary data, with a statistically significant *P* value of 0.018 (OR = 5.402, 95% CI = 1.329–21.959) suggesting that decrease in BMD value favoured the delayed group of treatment than the immediate. All the studies Z FAST, E-ZO FAST, and ZO FAST had a relative weight of 33.33, 36.51, and 30.17%, respectively. So, the contribution of each for getting an overall summary effect was relatively equal (Figure 2).

**Figure 3:**
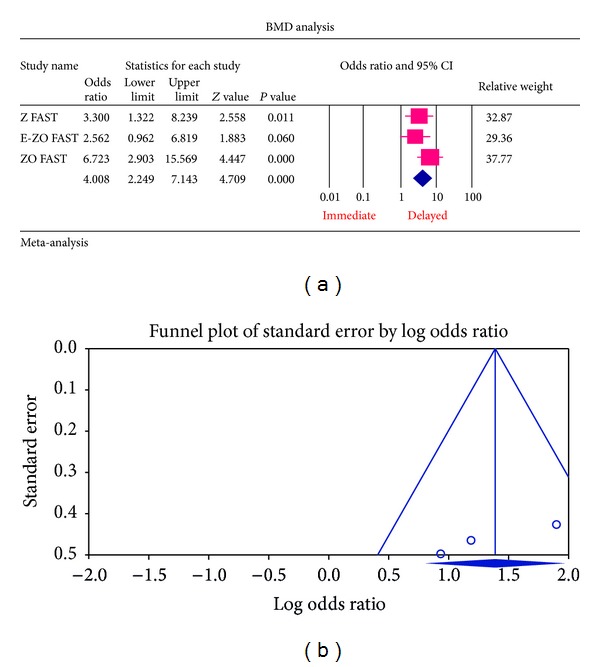
(a) Forest plot from the meta-analysis of LS BMD *T* score analysis of patients at 12 months, who had osteopenic BMD at baseline, between immediate and delayed zoledronic acid groups. (b) Funnel plot from the meta-analysis of LS BMD *T* score analysis of patients at 12 months, who had osteopenic BMD at baseline, between immediate and delayed zoledronic acid groups.

*(b) Patients with Osteopenic Bone Mineral Density at Baseline*. The overall summary statistics of patients having osteopenic BMD at baseline had a statistically significant *P* value of 0.0002 (OR = 4.008, 95% CI = 2.249–7.143) showing that decrease in BMD value is favoured in the delayed group than the immediate group (Figure 3).

#### 3.1.2. Bone Mineral Density Analysis for SABRE, ARIBON, and ARBI Trials

The meta-analysis of SABRE, ARIBON, and ARBI trials had a statistically significant result of *P* value of 0.0003 (standard difference in means 0.462, 95% CI = 0.270–0.654). SABRE trial contributed more weight to the overall summary effect (relative weight of 55.40) compared to ARIBON trial which had less relative weight of 16.84%. The results favoured the bisphosphonates + placebo group for decrease in BMD value compared with bisphosphonates + AIs, indicating that the bisphosphonates favour an increase in BMD.

### 3.2. Adverse Drug Reactions

#### 3.2.1. Delayed ZOL versus Immediate ZOL

The most common ADRs noted in the Z FAST, ZO FAST, and E-ZO FAST trials with zoledronic acid were bonepain, arthralgia, back pain, pain in extremities, and myalgia. However, none required the withdrawal of the drug. For bone pain, there was a statistically significant result for risk of not getting an ADR was significantly higher in the delayed group compared to the immediate group (OR = 2.177, 95% CI = 1.572–3.014, *P* value = 0.0002) (Figure 6). In arthralgia (Figure 5), there was no statistically significant difference in the risk of arthralgia between two groups (OR = 1.062, 95% CI = 0.886–1.272, *P* value = 0.5159). Similar results were identified for back pain, pain in extremity, and myalgia which are not statistically significant (OR = 0.878, 95% CI = 0.616–1.253, *P* value = 0.474; OR = 1.078, 95% CI = 0.615–1.887, *P* value = 0.794; OR = 1.168, 95% CI = 0.886–1.540, *P* value = 0.271, resp.) (Figures 7, 8, and 9 resp.).

#### 3.2.2. Publication Bias

**Figure 4:**
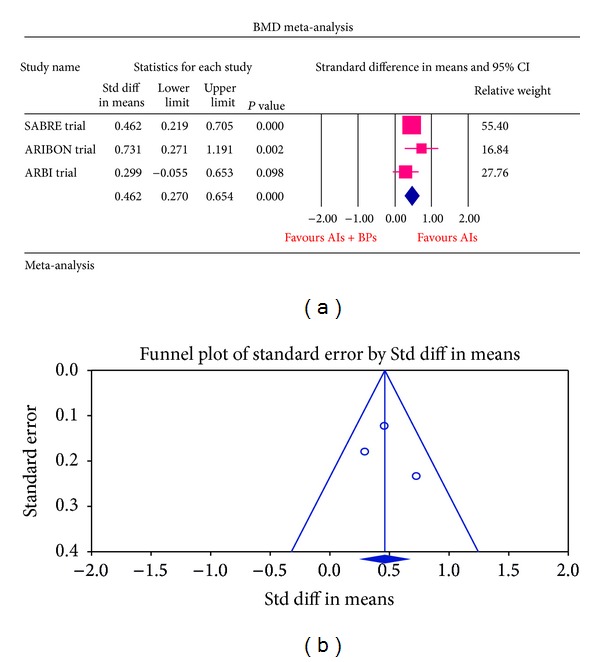
(a) Forest plot from meta-analysis of the BMD change of patients on aromatase inhibitors treated with bisphosphonates (ibandronate/risedronate) compared with aromatase inhibitors alone in osteopenic patients. (b) Funnel plot from meta-analysis of the BMD change of patients on aromatase inhibitors treated with bisphosphonates (ibandronate/risedronate) compared with aromatase inhibitors alone in osteopenic patients.

**Figure 5:**
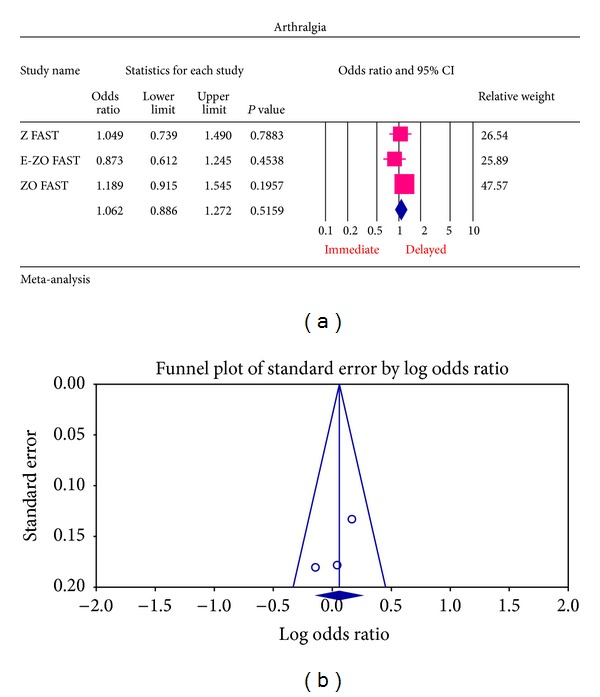
(a) Forest plot from meta-analysis of arthralgia incidence of patients treated with immediate zoledronic acid versus delayed zoledronic acid. (b) Funnel plot from meta-analysis of arthralgia incidence of patients treated with immediate zoledronic acid versus delayed zoledronic acid.

**Figure 6:**
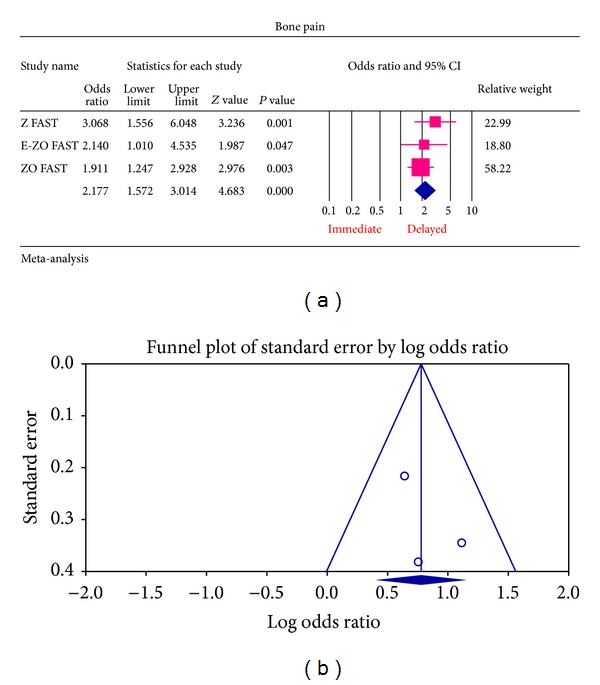
(a) Forest plot from meta-analysis of bone pain incidence of patients treated with immediate zoledronic acid versus delayed zoledronic acid. (b) Funnel plot from meta-analysis of bone pain incidence of patients treated with immediate zoledronic acid versus delayed zoledronic acid.

**Figure 7:**
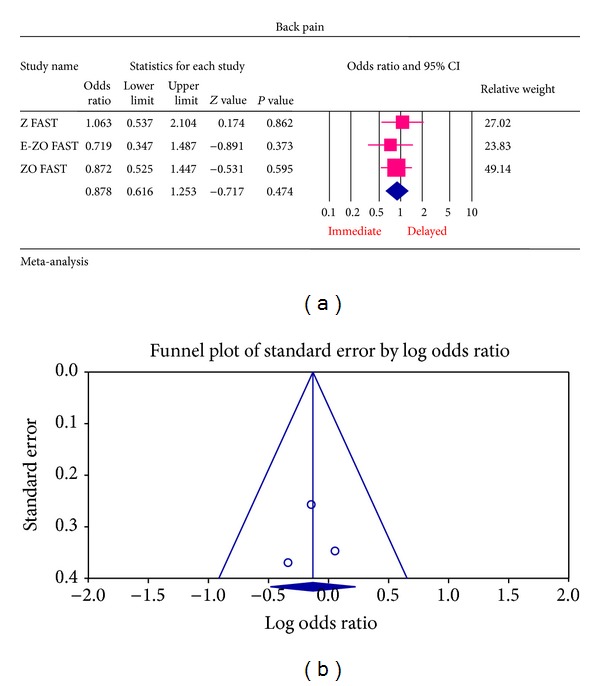
(a) Forest plot from meta-analysis of back pain incidence of patients treated with immediate zoledronic acid versus delayed zoledronic acid. (b) Funnel plot from meta-analysis of back pain incidence of patients treated with immediate zoledronic acid versus delayed zoledronic acid.

**Figure 8:**
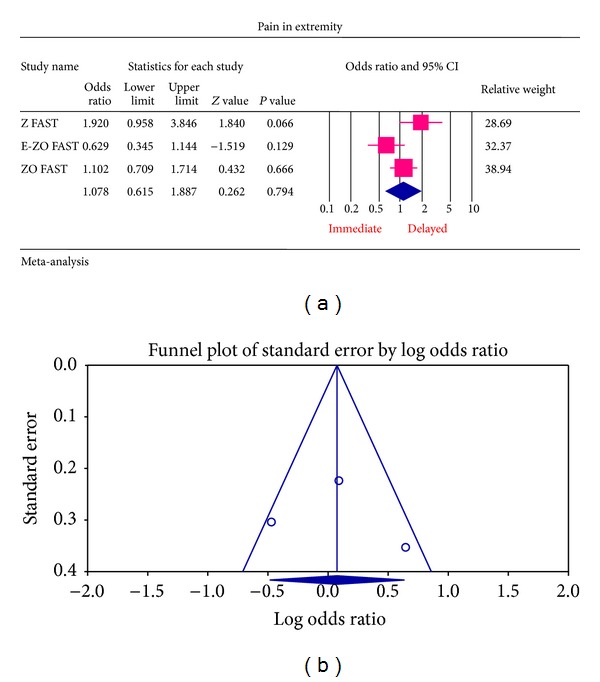
(a) Forest plot from meta-analysis of pain in extremity incidence of patients treated with immediate zoledronic acid versus delayed zoledronic acid. (b) Funnel plot from meta-analysis of pain in extremity incidence of patients treated with immediate zoledronic acid versus delayed zoledronic acid.

**Figure 9:**
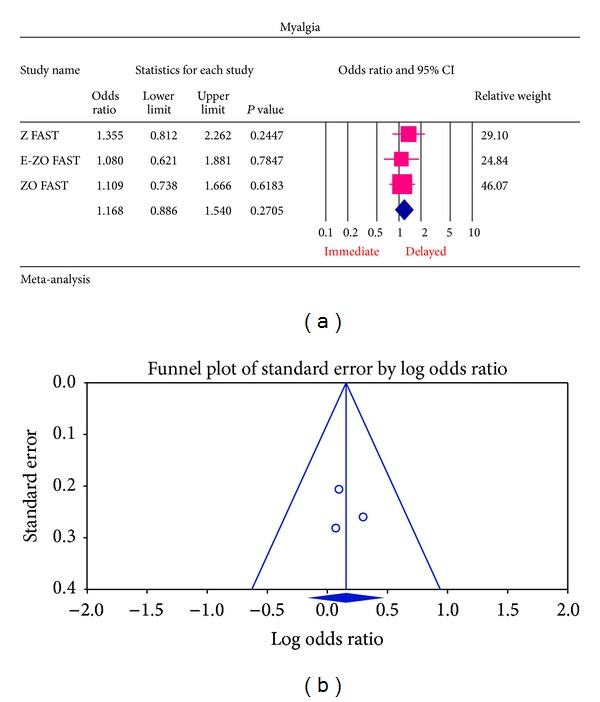
(a) Forest plot from meta-analysis of myalgia incidence of patients treated with immediate zoledronic acid versus delayed zoledronic acid. (b) Funnel plot from meta-analysis of pain in myalgia incidence of patients treated with immediate zoledronic acid versus delayed zoledronic acid.

For each of the different aspects of bisphosphonate use studied, no publication bias (Figures 3(a), 4(a), 5(a), 6(a), 7(a), 8(a), and 9(a)) was observed in funnel plots of these studies. Only in the case of bisphosphonate use in patients with normal BMD at baseline, publication bias was observed (Figure 2(a)). Sensitivity analysis was not done as the number of studies was small.

#### 3.2.3. Heterogeneity

Heterogeneity can be judged graphically, by looking at the forest plot, and can be measured statistically. From Table 5, all the *P* values are found statistically insignificant (*P* < 0.1). Among ADRs pain in extremity and from BMD analysis, 12-month LS BMD normal, at baseline, patients have a significant result.

## 4. Discussion

In this systematic review and meta-analysis, a total of 2772 postmenopausal patients with breast cancer were included. The pooled odds ratio and standard mean difference with 95% CI indicated that third-generation bisphosphonate therapy was associated with significant improvement in the BMD. For adverse events, we noted that in immediate group zoledronic acid therapy was associated with an increased risk of getting musculoskeletal adverse effects like arthralgia, myalgia, bone pain, back pain, and pain in extremity.

A study [21] suggested that the prevention of continuously decreasing BMD during endocrine treatment with aromatase inhibitors can be increased with the administration of bisphosphonates. Several clinical trials already proved that the combination of AIs with BPs has a potent effect on BMD.

Zoledronic acid which is a potent bisphosphonate has been shown to maintain or increase BMD in premenopausal women with early breast cancer or for those receiving adjuvant hormone therapies as well as healthy postmenopausal women [16, 17, 22]. The three trials Z FAST, ZO FAST, and E-ZO FAST are the zoledronic acid trials to evaluate i.v. bisphosphonates for the prevention of bone loss in these patients. These three trials evaluate the effect of upfront and delayed zoledronic acid for the prevention of bone loss in postmenopausal women with early breast cancer. These studies showed that upfront zoledronic acid is associated with increase in 12-month lumbar spine BMD compared with delayed bisphosphonates therapy. Studies of postmenopausal women with breast cancer receiving adjuvant AI therapy treated concurrently with an osteoblast inhibitor have shown the ability to maintain bone loss. Weekly oral alendronate (70 mg) or risedronate (35 mg), as well as monthly oral ibandronate (50 mg) and i.v. ibandronate (3 mg) once every 3 months, is considered as appropriate treatment for AI induced bone loss [23].

SABRE trial included in this study investigated the effects of adjuvant anastrazole with or without risedronate on BMD in postmenopausal women with hormone responsive early breast cancer and preexisting lower, moderate, and higher risk of fragility fracture. The trial further confirms that bone loss associated with anastrazole treatment is readily manageable with risedronate [18]. In another trial of risedronate and anastrazole therapy (ARBI trial) [20], it was found that there is a significant increase in BMD levels especially in high risk patients (*T* score of more than −2.0). The ARIBON trial evaluated the impact of oral ibandronate on BMD values and suggests it as effective treatment that could be considered for women with low BMD receiving aromatase inhibitor [19]. The meta-analysis of these studies also showed the same results, that is, increase in BMD, when the bisphosphonate (risedronate or ibandronate) is included in the treatment regimen of the patients.

Considering the secondary endpoint, that is, ADRs associated with bisphosphonates, a study on musculoskeletal disorders caused by zoledronic acid [24] concluded that patients treated with upfront ZOL had a significantly higher risk of bone pain than patients with delayed ZOL; in patients with a low risk of osteoporosis, immediate ZOL may not be needed due to additional adverse effects. These results are more or less similar to our study results on the occurrence of musculoskeletal ADRs.

Aromatase inhibitors used in hormone responsive breast cancer,tend to lower BMD scores, and the patient with osteoporosis also suffers serious musculoskeletal ADRs from zoledronic acid therapy. Zoledronic acid is mainly administered as intravenous infusions. To reduce these effects, protective measures like reducing the dose, slowing the infusion rate, and prolonging the interval between infusions should be considered. If the patients are not able to tolerate these ADRs, oral bisphosphonates should be considered [25]. Ibandronate is the only one new third-generation bisphosphonates that has developed in both intravenous and oral formulations for the management of bone disorders [26]. This study [26] also concluded in suggesting that oral ibandronate is of considerable clinical value to patients with painful metastases and also a useful coanalgesic to conventional treatment for malignant bone pain. However, they reported that 26.6% of the patients receiving oral ibandronate got upper gastrointestinal AEs.

There were similar studies of meta-analysis on bisphosphonate use in breast cancer patients. A study of Liu et al. which was conducted to quantify the risk reduction of breast cancer that is associated with bisphosphonate use and also to explore the treatment effect is based on duration of Bisphosphonates use exists and also concluded the fact that bisphosphonate use have a protective effect on breast cancer patients [27]. Another meta-analysis done by Huang et al. has given a precise estimate of musculoskeletal disorders of zoledronic acid in adjuvant breast cancer treatment [1]. But, these meta-analyses have chosen overall survival, disease free survival or recurrence free survival as their endpoints. In our meta-analysis, we have chosen bone mineral density as the primary endpoint that will give more precise estimate on bone health of breast cancer patients.

Denosumab is an antireceptor activator of nuclear factor- (NF-) kappa ligand (RANK) ligand human monoclonal antibody, which is also a treatment option for bone reduction in metastatic cancers. There are clinical trials comparing denosumab with zoledronic acid for efficacy and safety. These clinical trials discussed more about metastatic stage of breast cancer that affected the bones [28, 29]. Regarding efficacy both drugs appeared to have the same response. Patient compliance may not be a major issue both in case of denosumab (SC, once in 6 months) and also bisphosphonates (IV, once in 6 months), if they did not prefer oral therapy of bisphosphonates. Denosumab can be an alternative for BPs therapy only if the patient has renal insufficiency. If we compare the safety profiles of these two drugs, osteonecrosis of jaw was common in both cases and also acute phase reactions and flu-like symptoms are less common in BPs IV therapy [30]. However, when cost effectiveness is considered, the bisphosphonates are a better option than denosumab if renal function is normal.

There have been publications in the past four years, describing numerous cases of osteonecrosis of jaw, which is the most common and most discussed ADR by researchers, especially in patients affected with breast cancer or myeloma and also under treatment with pamidronate and zoledronic acid, and more rarely alendronate [30]. In our included studies of zoledronic acid, they have not mentioned osteonecrosis of jaw as their adverse event.

When the number of studies incorporated is small, publication bias is to be considered. The possibility of publication bias is always a concern. Of the 8 meta-analyses done on each subject for publication bias, only one study showed publication bias between the studies, that is, the 12-month LS BMD analysis of patients who had a normal BMD at baseline. The heterogeneity of clinical features such as race, age, number of study population, and study quality which are all also having fundamental importance to a meta-analysis were found to be within statistical limits. Although our systematic review included 6 RCTs, the subgroup meta-analysis was based on a smaller number of studies, and the evidence could have been stronger if the number was bigger. Only three RCTs are included in our meta-analysis for each subject. So, the findings should be carefully understood.

From the above analysis, it may be concluded that bisphosphonate has a positive role in the bone health of postmenopausal breast cancer patients on aromatase inhibitors and may be added to standard adjuvant therapy to improve the prognosis in postmenopausal patients.

## 5. Conclusion

 The findings of this study indicate that the third-generation bisphosphonates have positive effect on bone mineral density of patients who are suspected to or have suffered from bone loss, osteopenia, or osteoporosis due to the treatment of aromatase inhibitors in hormone responsive breast cancer. Furthermore, the patients treated with immediate zoledronic acid had a significantly higher risk of musculoskeletal ADRs than patients with delayed zoledronic acid.

Suggestion from the meta-analysis is that measuring the bone mineral density at intermittent intervals of time throughout the therapeutic regimen should be recorded and reported and bisphosphonates therapy should be started accordingly. This can avoid further risk of bone loss, bone fractures, or osteoporosis conditions due to treatment with aromatase inhibitors in breast cancer. Second suggestion is that adverse effects in clinical trials and otherwise should be recorded and reported so that they can be evaluated in future trials and risk and benefit can be judged.

## Figures and Tables

**Table 1:** Evidence-based Approach.

PICO statement	
Patient/problem (P)	Postmenopausal breast cancer patients/low BMD *T* score, musculoskeletal ADRs
Intervention (I)	Third-generation BPs and AIs
Comparison (if any) (C)	Immediate and delayed therapy/placebo
Outcome (O)	Increase in BMD *T* score
Type of question	Therapy
Type of study	Randomised control trials

**Table 2:** Design and characteristics of trials included in the systematic review and metaanalysis.

Author (trial)	Year	Number of patients	Dosage of bisphosphonate	Combination therapy	Duration	Median follow up (months)	Intervention	Jadad score
Lombart et al. (E-ZO FAST)	2009	527	4 mg IV every 6 months	2.5 mg letrozole daily	5 years	36	Immediate ZOL 4 mg every 6 months, delayed ZOL 4 mg every 6 months	3
Bundred et al. (ZO FAST) [17]	2008	1065	4 mg IV every 6 months	2.5 mg letrozole daily	5 years	48	Immediate ZOL 4 mg every 6 months, delayed ZOL 4 mg every 6 months	4
Brufsky et al. (Z FAST) [16]	2007	602	4 mg IV every 6 months	2.5 mg letrozole daily	5 years	54	Immediate ZOL 4 mg every 6 months, delayed ZOL 4 mg every 6 months	4
Van Poznak et al. (SABRE trial) [18]	2010	234	Residronate 35 mg per week	1 mg Anastrozole daily	2 years	24	Anastrozole + residronate, anastrozole + placebo	4
Markopoulos et al. (ARBI trial) [20]	2010	213	Residronate 35 mg per week	1 mg Anastrozole daily	3 years	36	Anastrozole + residronate, anastrozole + placebo	3
Lester et al. (ARIBON trial) [19]	2008	131	Ibandronate 150 mg every 28 day	1 mg Anastrozole daily	2 years	24	Anastrazole + ibandronate, anastrozole + placebo	3

**Table 3:** Patient characteristics of included zoledronic acid trials.

Characteristic	Immediate group			Delayed group		
	Z FAST	ZO FAST	E-ZO FAST	Z FAST	ZO FAST	E-ZO FAST
Age, years						
Median	60	57	58	60	58	58
Range	35–83	36–87	40–81	41–89	37–81	44–78
Race						
White	280	—	226	269	—	242
Asian	—	89	21	—	93	19
Black	9	—	—	14	—	—
Caucasian	—	416	—	—	409	—
Other	12	27	5	18	31	9
Baseline *T* score						
*T* score > −1	217	370	163	216	367	180
*T* score ≤ −1 to ≥ −2	84	162	89	85	166	90
Postmenopausal status						
Postmenopausal	281	445	210	284	443	228
Recently postmenopausal	—	87	42	—	90	42
Bone mineral density						
Lumbar spine						
Mean	1.110	1.08		1.106	1.08	
SD	0.1652	0.16		0.1663	0.16	
Median	1.088	1.07	—	1.082	1.06	—
Range	0.818–1.649	0.79–1.67		0.807–1.692	0.71–1.77	
Total hip						
Mean	0.958	0.96		0.955	0.96	
SD	0.1259	0.13	—	0.1322	0.13	—
Median	0.954	0.94		0.943	0.94	
Range	0.676–1.310	0.62–1.43		0.700–1.475	0.62–1.55	

**Table 4:** Adverse events occurring in greater than 5% of patients in Z FAST, ZO FAST, and E-ZO FAST trials.

Adverse event	Number of patients in immediate ZOL			Number of patients in delayed ZOL		
	Z FAST	ZO FAST	E-ZO FAST	Z FAST	ZO FAST	E-ZO FAST
Arthralgia	90	172	90	87	152	105
Myalgia	38	54	28	29	49	28
Bone pain	34	65	21	12	36	11
Pain in extremity	24	45	19	13	45	31
Back pain	18	30	13	17	30	19

**Table 5:** Heterogeneity test results.

Outcome	*P* value
Arthralgia	0.390
Bone pain	0.608
Back pain	0.744
Pain in extremity	0.056
Myalgia	0.794
LS BMD 12 months (normal at baseline-ZOL trials)	0.0003
LS BMD 12 months (osteopenic at baseline-ZOL trials)	0.296
LS BMD 12 months (residronate and ibandronate trials)	0.345

## References

[1] Huang WW, Huang C, Liu J, Zheng HY, Lin L (2012). Zoledronic acid as an adjuvant therapy in patients with breast cancer: a systematic review and meta-analysis. *PloS ONE*.

[2] Iwata H, Masuda N, Ohno S (2013). A randomized, double-blind, controlled study of exemestane versus anastrozole for the first-line treatment of postmenopausal Japanese women with hormone-receptor-positive advanced breast cancer. *Breast Cancer Research and Treatment*.

[3] Eastell R, Hannon RA, Cuzick J, Dowsett M, Clack G, Adams JE (2006). Effect of an aromatase inhibitor on BMD and bone turnover markers: 2-Year results of the anastrozole, tamoxifen, alone or in combination (ATAC) trial (18233230). *Journal of Bone and Mineral Research*.

[4] Eastell R, Adams JE, Coleman RE (2008). Effect of anastrozole on bone mineral density: 5-year results from the anastrozole, tamoxifen, alone or in combination trial 18233230. *Journal of Clinical Oncology*.

[5] Lin NU, Winer EP (2008). Advances in adjuvant endocrine therapy for postmenopausal women. *Journal of Clinical Oncology*.

[6] Rabaglio M, Sun Z, Price KN (2009). Bone fractures among postmenopausal patients with endocrine-responsive early breast cancer treated with 5 years of letrozole or tamoxifen in the BIG 1-98 trial. *Annals of Oncology*.

[7] Simpson ER, Dowsett M (2002). Aromatase and its inhibitors: significance for breast cancer therapy. *Recent Progress in Hormone Research*.

[8] Geisler J, Lønning PE (2005). Endocrine effects of aromatase inhibitors and inactivators in vivo: review of data and method limitations. *Journal of Steroid Biochemistry and Molecular Biology*.

[9] Coleman RE, Body J-J, Gralow JR, Lipton A (2008). Bone loss in patients with breast cancer receiving aromatase inhibitors and associated treatment strategies. *Cancer Treatment Reviews*.

[10] Coleman RE (2005). Bisphosphonates in breast cancer. *Annals of Oncology*.

[11] Kimmel DB (2007). Mechanism of action, pharmacokinetic and pharmacodynamic profile, and clinical applications of nitrogen-containing bisphosphonates. *Journal of Dental Research*.

[12] Steves R, Hootmant JM (2004). Evidence-based medicine: what is it and how does it apply to athletic training?. *Journal of Athletic Training*.

[13] Akobeng AK (2005). Principles of evidence based medicine. *Archives of Disease in Childhood*.

[14] Jadad AR, Moore RA, Carroll D (1996). Assessing the quality of reports of randomized clinical trials: is blinding necessary?. *Controlled Clinical Trials*.

[15] Llombart A, Frassoldati A, Paija O (2012). Immediate administration of zoledronic acid reduces aromatase inhibitorassociated bone loss in postmenopausal women with early breast cancer: 12-month analysis of the E-ZO-FAST trial. *Clinical Breast Cancer*.

[16] Brufsky A, Harker WG, Beck JT (2007). Zoledronic acid inhibits adjuvant letrozole-induced bone loss in postmenopausal women with early breast cancer. *Journal of Clinical Oncology*.

[17] Bundred NJ, Campbell ID, Davidson N (2008). Effective inhibition of aromatase inhibitor-associated bone loss by zoledronic acid in postmenopausal women with early breast cancer receiving adjuvant letrozole: ZO-FAST study results. *Cancer*.

[18] van Poznak C, Hannon RA, Mackey JR (2010). Prevention of aromatase inhibitor-induced bone loss using risedronate: the SABRE trial. *Journal of Clinical Oncology*.

[19] Lester JE, Dodwell D, Purohit OP (2008). Prevention of anastrozole-induced bone loss with monthly oral ibandronate during adjuvant aromatase inhibitor therapy for breast cancer. *Clinical Cancer Research*.

[20] Markopoulos C, Tzoracoleftherakis E, Polychronis A (2010). Management of anastrozole-induced bone loss in breast cancer patients with oral risedronate: results from the ARBI prospective clinical trial. *Breast Cancer Research*.

[21] Gnant MFX, Mlineritsch B, Luschin-Ebengreuth G (2007). Zoledronic acid prevents cancer treatment-induced bone loss in premenopausal women receiving adjuvant endocrine therapy for hormone-responsive breast cancer: a report from the Austrian Breast and Colorectal Cancer Study Group. *Journal of Clinical Oncology*.

[22] Gnant M, Mlineritsch B, Luschin-Ebengreuth G (2008). Adjuvant endocrine therapy plus zoledronic acid in premenopausal women with early-stage breast cancer: 5-year follow-up of the ABCSG-12 bone-mineral density substudy. *The Lancet Oncology*.

[23] Reid DM, Doughty J, Eastell R (2008). Guidance for the management of breast cancer treatment-induced bone loss: a consensus position statement from a UK Expert Group. *Cancer Treatment Reviews*.

[24] Zhou W-B, Zhang P-L, Liu X-A, Yang T, He W (2011). Innegligible musculoskeletal disorders caused by zoledronic acid in adjuvant breast cancer treatment: a meta-analysis. *Journal of Experimental & Clinical Cancer Research*.

[25] Diel IJ, Bergner R, Grötz KA (2007). Adverse effects of bisphosphonates: current issues. *Journal of Supportive Oncology*.

[26] Body JJ, Diel IJ, Liehinitzer M (2004). Oral ibandronate reduces the risk of skeletal complications in breast cancer patients with metastatic bone disease: results from two randomised, placebo-controlled phase III studies. *British Journal of Cancer*.

[27] Liu Y, Zhao S, Chen W (2012). Bisphosphonates use and the risk of breast cancer : a meta-analysis of published literature. *Clinical Breast Cancer*.

[28] Stopeck AT, Lipton A, Body J-J (2010). Denosumab compared with zoledronic acid for the treatment of bone metastases in patients with advanced breast cancer: a randomized, double-blind study. *Journal of Clinical Oncology*.

[29] Fizazi K, Lipton A, Mariette X (2009). Randomized phase II trial of denosumab in patients with bone metastases from prostate cancer, breast cancer, or other neoplasms after intravenous bisphosphonates. *Journal of Clinical Oncology*.

[30] Agarwal P, Rao NN (2012). Bisphosphonates associated osteonecrosis of the jaws. *Indian Journal of Dental Research*.

